# Insulin-like growth factor-I induces epithelial to mesenchymal transition via GSK-3β and ZEB2 in the BGC-823 gastric cancer cell line

**DOI:** 10.3892/ol.2014.2687

**Published:** 2014-11-07

**Authors:** HEMING LI, LING XU, LEI ZHAO, YANJU MA, ZHITU ZHU, YUNPENG LIU, XIUJUAN QU

**Affiliations:** 1 Department of Medical Oncology, The First Hospital of China Medical University, Shenyang, Liaoning 110001, P.R. China; 2 Department of Oncology, The First Affiliated Hospital of Liaoning Medical University, Jinzhou, Liaoning 121001, P.R. China

**Keywords:** epithelial-to-mesenchymal transition, insulin-like growth factor-I, insulin-like growth factor-I receptor, ZEB2, GSK-3β

## Abstract

Metastasis is the most common cause of mortality in patients with gastric cancer. Epithelial-to-mesenchymal transition (EMT), which may be stimulated by insulin-like growth factor-I (IGF-I) is involved in the metastasis of numerous tumors; however, the molecular mechanism by which IGF-I may induce tumor cell EMT remains to be elucidated in gastric cancer. The present study aimed to investigate the induction of EMT in BGC-823 gastric cancer cells. It was identified that IGF-I induced EMT by upregulating the levels of ZEB2 transcription factor, and this was dependent on the phosphoinositide 3-kinase (PI3K)/Akt signaling pathway in these cells. In addition, glycogen synthase kinase 3β (GSK-3β), an intracellular downstream effector of PI3K/Akt, sustained the epithelial phenotype by repressing ZEB2 expression and the subsequent inhibition of EMT induced by IGF-I, suggesting the involvement of a potential PI3K/Akt-GSK-3β-ZEB2 signaling pathway in IGF-I-induced EMT in gastric cancer BGC-823 cells. Overall, the results of the present study suggest that IGF-I induced EMT by the activation of a PI3K/Akt-GSK-3β-ZEB2 signaling pathway in gastric cancer BGC-823 cells. Therefore, this study may provide more useful information regarding the mechanism of gastric cancer metastasis.

## Introduction

Gastric cancer is one of the most common types of cancer worldwide and the predominant cause of cancer-related mortality in Asian countries annually (1,2). The majority of patients are diagnosed with advanced disease, particularly in China (3). Advanced gastric cancer is characterized by a highly invasive and metastatic malignancy (3). Although chemotherapy, radiotherapy and targeted therapy have improved the response rate in advanced gastric cancer patients, metastasis remains the most common cause of mortality (4). Contributing to this problem is the unclear metastatic mechanism and the lack of effective biomarkers for metastasis prediction. Therefore, investigations on the molecular mechanisms of tumor metastasis will provide potential molecular targets and biomarkers for the development of effective therapies for gastric cancer.

Numerous different processes are involved in tumor metastasis. Growing evidence indicates that epithelial-to-mesenchymal transition (EMT) is a major contributor to tumor metastasis in several types of epithelial tumor cells (5,6). EMT is a developmental process by which non-motile epithelial cells characterized of cell-cell tight conjunctions lose their epithelial polarity and become migratory mesenchymal cells (7). The insulin-like growth factor-I (IGF-I) axis has been reported to induce EMT and promote tumor metastasis in prostate and breast cancer cells (8–10). Meanwhile, basic and clinical studies have reported that overexpression of IGF-I receptor (IGF-IR) is associated with enhanced invasiveness in gastrointestinal tumor cells, and with poor survival in gastric cancer patients (11–13). However, two recent phase II and III clinical trials reported that monoclonal antibodies targeting the IGF-IR did not improve overall survival in breast and pancreatic cancer patients, respectively (14,15). This failure follows a slew of setbacks for IGF-IR-targeted therapies. Therefore, exploring the mechanisms of IGF-I-induced tumor metastasis, finding predictive biomarkers and selecting suitable patients appear to be important for further development of IGF-IR-specific targeting therapy.

The phosphoinositide 3-kinase (PI3K)/Akt pathway is downstream of IGF-I/IGF-IR signaling, and contains important signaling molecules in the regulation of IGF-IR-mediated EMT (16–18). Among these effectors, glycogen synthase kinase 3β (GSK-3β) is a multifunctional kinase capable of being inactivated by Akt (19,20). Previous studies have reported that GSK-3β maintains epithelial phenotypes and inhibits migration in certain types of epithelial tumor cells (21,22). Consistently, GSK-3β can also repress IGF-IR-mediated EMT in breast epithelial cells (16). However, the specific effects of GSK-3β in IGF-I-induced gastric cancer EMT remain unclear.

Consequently, in the present study, the direct role of IGF-I in promoting EMT in gastric cancer and the role of the PI3K/Akt signaling pathway, GSK-3β and ZEB2 in this process was investigated.

## Materials and methods

### Cell culture

The BGC823 human gastric cell line was obtained from the Cell Bank of Type Culture Collection of the Chinese Academy of Sciences (Shanghai, China). The cells were maintained in RPMI-1640 medium (Gibco-BRL, Carlsbad, CA, USA) supplemented with 10% fetal bovine serum (Gibco-BRL), penicillin (100 U/ml; Invitrogen Life Technologies, Inc., Carlsbad, CA, USA) and streptomycin (100 mg/ml; Invitrogen Life Technologies, Inc.) in a humidified atmosphere of 5% CO_2_ and 95% air, at 37°C. The cells were serum-starved overnight before human recombinant IGF-I (100 ng/ml; R&D Systems, Wiesbaden, Germany) treatment. All the cells used for the experiments were subcultured every 2–3 days and harvested in the logarithmic phase of growth.

### Reagents and antibodies

IGF-I was purchased from R&D Systems, while specific PI3K/Akt inhibitor, LY294002, and GSK-3β inhibitor, AR-A01448, were purchased from Sigma-Aldrich (St. Louis, MO, USA). The dual IGF-IR/IR inhibitor, OSI-906, was purchased from SelleckBio (Houston, TX, USA). Monoclonal rabbit anti-human E-cadherin, monoclonal rabbit anti-human vimentin, monoclonal rabbit anti-human IGF-IR, monoclonal rabbit anti-human ZEB1, monoclonal rabbit anti-human GSK-3β, polyclonal rabbit anti-human phospho-IGF-IR (Tyr1131) and monoclonal rabbit anti-human phospho-GSK-3β (Ser9) antibodies were purchased from Cell Signaling Technology, Inc. (Beverly, MA), while polyclonal rabbit anti-human Twist2 antibody was purchased from Abcam (Cambridge, MA, USA). Monoclonal mouse anti-human ZEB2, monoclonal rabbit anti-human Twist1, polyclonal rabbit anti-human actin, monoclonal rabbit anti-human Akt and polyclonal rabbit anti-human phospho-Akt (Ser473) antibodies were purchased from Santa Cruz Biotechnology, Inc. (Santa Cruz, CA, USA).

### Western blot assay

Cells were washed three or four times with 1X phosphate-buffered saline (PBS), solubilized in 1% Triton lysis buffer [50 mM Tris-HCl (pH 7.4), 150 mM NaCl, 10 mM EDTA, 100 mM NaF, 1 mM Na_3_VO_4_, 1% Triton X-100 (Sigma-Aldrich), 1 mM PMSF and 2 μg/ml aprotinin] on ice, and then quantified according to the Lowry method (23). Following this, all the samples were eluted by boiling water at 100°C for 5 min with 3X sampling buffer. Total proteins were subjected to sodium dodecyl sulfate-polyacrylamide gel electrophoresis and electronically transferred to nitrocellulose membranes. The blots were incubated with E-cadherin, vimentin, IGF-IR, ZEB1/2, Twist1/2, GSK-3β, Akt, actin, phospho-IGF-IR (Tyr1131), phospho-GSK-3β(Ser9) or phospho-Akt (Ser473) antibodies at 4°C overnight, after blocking with 5% skimmed milk in TBST [10 mM Tris (pH 7.4), 150 mM NaCl and 0.1% Tween-20]. On the following day, the blots were incubated with monoclonal anti-rabbit or mouse secondary antibodies (Santa Cruz Biotechnology, Inc.) for 30 min at room temperature. After four washes with TBST, proteins were detected using an enhanced chemiluminescence reagent (SuperSignal Western Pico Chemiluminescent Substrate; Pierce, Rockford, IL, USA) and visualized with an enhanced chemiliuminescence detection system (DNR Bio-Imaging Systems, Ltd., Jerusalem, Israel). The images were then analyzed by National Institutes of Health image software (http://rsb.info.nih.gov/nih-image/) for further statistical analysis.

### Immunofluorescence

The cells (2×10^4^ cells/well) were seeded in Lab-Tek chamber slides (Nunc S/A; Polylabo, Strasbourg, France). After being serum-starved overnight, the cells were treated with or without IGF-I (100 ng/ml) for 48 h and fixed with 3.3% paraformaldehyde for 15 min, followed by rinsing with 1X PBS three times at room temperature. For morphological analysis, cells were permeabilized with 0.2% Triton X-100 for 5 min, blocked with 5% bovine serum albumin (Sigma-Aldrich) in 1X PBS for 1 h at room temperature, and then incubated with anti-E-cadherin or anti-Vimentin antibody overnight at 4°C. On the next day, alexa Fluor 546-conjugated goat monoclonal anti-rabbit IgG or Alexa Fluor 488-conjugated goat monoclonal anti-rabbit IgG (Molecular Probes, Eugene, OR, USA) were added in blocking solution for 1 h at room temperature in the dark. 4′6-diamidino-2-phenylindole was used to stain nuclei for 5 min. The cells were visualized by fluorescence microscopy (BX61; Olympus, Tokyo, Japan) following mounting using the SlowFade Antifade kit (Molecular Probes).

### Statistical analysis

All data presented in the study are expressed as the mean ± standard deviation. Representative results were from at least three independent experiments. Significant differences between treated and control groups were calculated using the two-tailed Student’s t-test. P<0.05 was considered to indicate a statistically significant difference. Statistical analysis was performed using SPSS version 18.0 software (SPSS, Inc., Chicago, IL, USA).

## Results

### IGF-I induces EMT in BGC-823 gastric cancer cells

**Figure 1:**
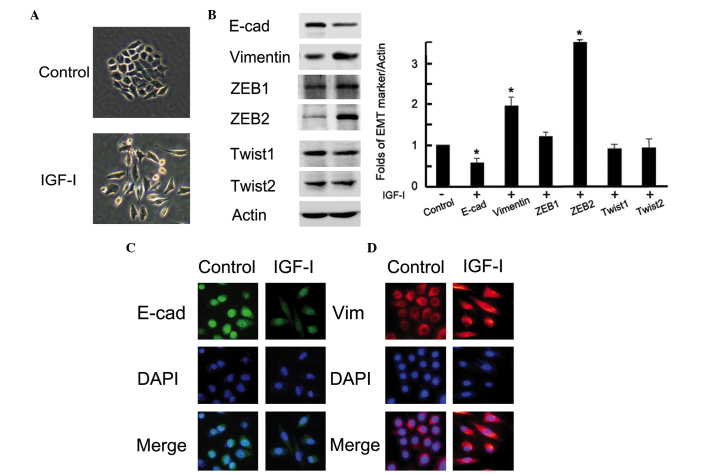
IGF-I induced epithelial-to-mesenchymal transition in BGC-823 gastric cancer cells. BGC-823 cells were serum-starved overnight and then treated with or without 100 ng/ml IGF-I for 48h. (A) Photographs were taken at ×20 magnification. (B) Cell lysates were collected for western blot analysis. (C and D) The cells were stained with antibodies to E-cadherin (green) and vimentin (red), and nuclei were stained with 4′,6′-diamidino-2-phenylindole (blue). Images were captured by fluorescence microscopy at ×40 magnification. IGF-I, insulin-like growth factor-I; E-cad, E-cadherin; Vim, Vimentin.

To investigate the role of IGF-I in gastric cancer cells, we treated BGC-823 cells with human recombinant IGF-I (100 ng/ml) for 48 h after overnight serum starvation. As shown in Fig. 1A, the cells with IGF-I treatment represented a mesenchymal phenotype; a loss of tight cell-cell junctions, an increase in cell scattering and an elongation of the cell shape. These morphological changes were compatible with the characteristics of EMT. Following IGF-I treatment, both western blotting and immunofluorescence observed obvious EMT marker switching, as shown by the downregulation of the epithelial marker E-cadherin and the upregulation of the mesenchymal marker vimentin in BGC-823 gastric cancer cells (P<0.05; Fig. 1B and C). In addition, the expression of the transcription factor ZEB2 was markedly upregulated after IGF-I treatment (P=0.01); however, the expression of other measured proteins, ZEB1, Twist1 and Twist2, did not change (P<0.05; Fig. 1B). These data demonstrated that IGF-I could upregulate ZEB2 and induce EMT in BGC-823 gastric cancer cells.

### Activation of the PI3K/Akt downstream pathway is required for IGF-I-induced upregulation of ZEB2

**Figure 2:**
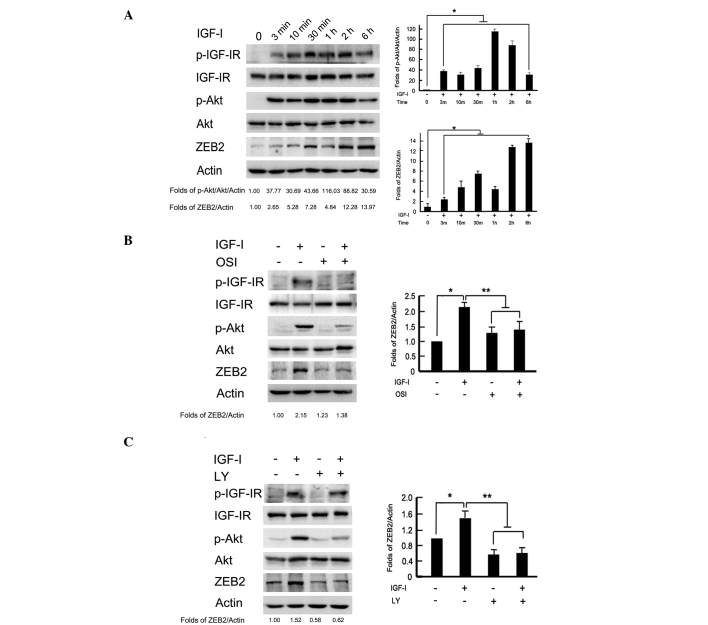
Activation of the PI3K/Akt downstream pathway was required for IGF-I-induced ZEB2 upregulation. (A) BGC-823 cells were incubated with IGF-I (100 ng/ml) for the indicated time periods, and the phosphorylation of IGF-IR and Akt, as well as ZEB2 expression, were analyzed by western blotting. The serum-starved cells were pretreated with or without (B) IGF-IR inhibitor, OSI-906 (10 μM) or (C) PI3K/Akt inhibitor, LY294002 (100 μM) for 2 h, followed by IGF-I (100 ng/ml) stimulation for 1 h. Cell lysates were collected for western blot analysis. PI3K, phosphoinositide 3-kinase; IGF-I, insulin-like growth factor-I; OSI, OSI-906; LY, LY294002.

Given that PI3K/Akt is a downstream signaling pathway of IGF-I/IGF-IR, we further examined the activation level of this signaling pathway. As shown in Fig. 2A, the phosphorylation levels of IGF-IR and Akt were time-dependently increased by IGF-I stimulation. Transient phosphorylation of IGF-IR and Akt was detected at 3 min to 2 h, and gradually recovered to the baseline values following exposure to IGF-I for 6 h. Meanwhile, IGF-I stimulation time-dependently upregulated ZEB2 expression. To analyze whether ZEB2 activation was dependent on the PI3K/Akt downstream pathway, BGC-823 cells were pretreated with IGF-IR inhibitor, OSI-906 (10 μM) and PI3K/Akt inhibitor, LY294002 (100 μM) 2 h to block the signaling pathways prior to IGF-I treatment. The protein levels of ZEB2 were upregulated after IGF-I treatment for 2 h. Pretreatment with OSI-906 or LY294002 markedly reversed the ZEB2 upregulation induced by IGF-I (P<0.05; Fig. 2B and C). These data indicated that IGF-I-induced ZEB2 upregulation was dependent on the PI3K/Akt downstream signaling pathway in BGC-823 gastric cancer cells.

### Activation of the PI3K/Akt downstream pathway is necessary for IGF-I-induced EMT

**Figure 3:**
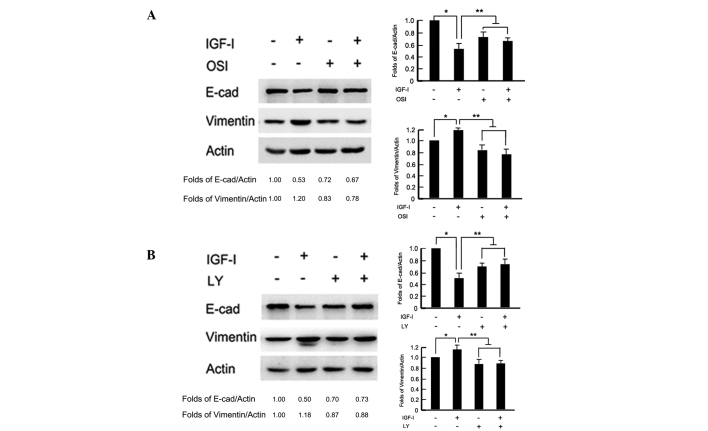
Activation of the PI3K/Akt downstream pathway was necessary for IGF-I-induced epithelial-to-mesenchymal transition. The serum-starved BGC-823 cells were pretreated with or without (A) IGF-IR inhibitor, OSI-906 (10 μM) or (B) PI3K/Akt inhibitor, LY294002 (100 μM) for 2 h, followed by IGF-I (100 ng/mL) stimulation for 48 h. Cell lysates were collected for western blot analysis. PI3K, phosphoinositide 3-kinase; IGF-I, insulin-like growth factor-I; E-cad, E-cadherin; OSI, OSI-906; LY, LY294002.

To test whether the PI3K/Akt pathway was involved in IGF-I-induced EMT, cells were pretreated with the IGF-IR inhibitor, OSI-906 (10 μM) and PI3K/Akt inhibitor, LY294002 (100 μM) 2 h before IGF-I stimulation for 48 h, respectively. In the presence of OSI-906, the BGC-823 cells maintained an epithelial like morphology with tight cell-cell junctions, following IGF-I treatment for 48 h (data not shown). Western blot analysis revealed that OSI-906 reversed IGF-I-induced E-cadherin downregulation and vimentin upregulation (P<0.05; Fig. 3A). Similarly, blocking the downstream signaling pathway with LY294002 repressed IGF-I-induced cellular morphology changes and attenuated EMT-associated marker, E-cadherin and vimentin, expression changes (P<0.05; Fig. 3B). These results indicated that the PI3K/Akt downstream pathway was necessary for IGF-I-induced EMT in BGC-823 gastric cancer cells.

### A potential PI3K/Akt-GSK-3β-ZEB2 signaling pathway is involved in IGF-I-induced EMT

**Figure 4:**
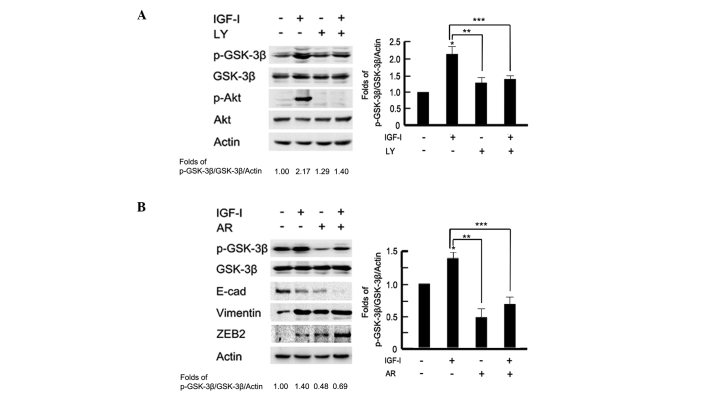
A PI3K/Akt-GSK-3β-ZEB2 signaling pathway is involved in IGF-I-induced epithelial-to-mesenchymal transition. The serum-starved BGC-823 cells were pretreated with or without (A) PI3K/Akt inhibitor, LY294002 (100 μM) or (B) GSK-3β inhibitor, AR-A01448 (25 μM) for 2 h, followed by IGF-I (100 ng/ml) treatment for 48 h. Cell lysates were collected for western blot analysis. The images were analyzed using NIH Image software and presented as histograms. Data are presented as the mean ± standard deviation of three independent experiments. ^*^IGF-I untreated vs. IGF-I treated, P<0.05. ^**^IGF-I treated vs. LY or AR treated, p < 0.05. *** IGF-I treated vs. IGF-I combined with LY or AR, P<0.05. The control group was used as the reference. PI3K, phosphoinositide 3-kinase; IGF-I, insulin-like growth factor-I; GSK-3β, glycogen synthase kinase 3β; E-cad, E-cadherin; LY, LY294002; AR, AR-A01448.

A previous study has reported that IGF-IR can modulate GSK-3β activity via Akt in MCF-10A cells (16). To test the effect of PI3K/Akt on GSK-3β expression, cells were pretreated with the PI3K/Akt inhibitor, LY294002 (100 μM) for 2 h prior to IGF-I stimulation. As shown in Fig. 4A western blot analysis and associated histograms indicated that pretreatment with LY294002 significantly inhibited the phosphorylation levels of GSK-3β following IGF-I stimulation (P<0.05). To further examine the role of GSK-3β in IGF-I-mediated EMT, BGC-823 cells were pretreated with the GSK-3β inhibitor, AR-A01448 (25 μM) for 2 h prior to IGF-I treatment for 48 h. Cells were observed to exhibit a mesenchymal phenotype in the AR-A01448-treated group; a loss of cell-cell contacts, an elongated cell shape and scattering of cells were evident (data not shown). Western blotting detected marked epithelial-mesenchymal phenotype marker switching. In addition, downregulation of E-cadherin and upregulation of vimentin and ZEB2 were also observed in AR-A01448-treated cells following IGF-I stimulation (P<0.05; Fig. 4B). These data indicated that there may be a PI3K/Akt-GSK-3β-ZEB2 signaling pathway involved in IGF-I-induced EMT in BGC-823 gastric cancer cells.

## Discussion

As previously reported, transcription factors ZEB1 and Snail are critical transcriptional regulators of IGF-I/IGF-IR-mediated EMT, and ultimately function as metastasis promoters through the repression of cell adhesion molecule E-cadherin in prostate and breast cancer cells (8–10). ZEB2 is another member of the ZEB family, and is a zinc finger protein with similar repressor effects to ZEB1 in terms of E-cadherin transcription (24,25). The ZEB2/E-cadherin ratio has been reported to be positively associated with tumor invasiveness and poor prognosis in breast and ovarian cancer (26). Transforming growth factor-β-mediated ZEB2 expression has been shown to be involved in the process of EMT and enhancement of invasive ability in several types of epithelial tumor cells (27,28); however, whether IGF-I can upregulate the expression of ZEB2 is yet to be elucidated. In the present study, it was observed that IGF-I induced EMT and upregulated ZEB2, but not ZEB1, Twist1 or Twist2, in gastric cancer BGC-823 cells. Furthermore, inhibition of the PI3K/Akt signaling pathway reversed ZEB2 upregulation and the subsequent EMT procession mediated by IGF-I. The results indicated that IGF-I induced EMT by upregulating ZEB2 expression, which was due to activating the downstream PI3K/Akt signaling pathway in BGC-823 gastric cancer cells.

To understand the mechanism by which the PI3K/Akt signaling pathway mediates ZEB2 expression, other intracellular downstream effectors of Akt were examined. A previous study has reported that GSK-3β, a major downstream component of the PI3K/Akt signaling pathway, is involved in IGF-IR-mediated EMT through a GSK-3β-NF-κB-Snail signaling pathway in immortalized mammary epithelial MCF10A cells (16). The present study demonstrated that GSK-3β maintained the epithelial phenotype of BGC-823 gastric cancer cells, and PI3K/Akt-GSK-3β signaling is an upstream factor of ZEB2 activation in the IGF-I-induced EMT process. These data indicated that a PI3K/Akt-GSK-3β-ZEB2 signaling pathway, which is involved in IGF-I-induced EMT, may exist in BGC-823 gastric cancer cells.

Overall, to the best of our knowledge, the present study is the first to report that IGF-I induces EMT, thereby upregulating ZEB2 expression, and that a potential PI3K/Akt-GSK-3β-ZEB2 signaling pathway is involved in IGF-I-induced EMT in BGC-823 gastric cancer cells. These results may be helpful for elucidating the mechanisms of IGF-I-mediated EMT procession. ZEB2 may serve as a clinical biomarker to identify patients who can benefit from IGF-IR-targeted therapy in gastric cancer.
